# Xiaoyaosan ameliorates depressive-like behavior and susceptibility to glucose intolerance in rat: involvement of LepR-STAT3/PI3K pathway in hypothalamic arcuate nucleus

**DOI:** 10.1186/s12906-023-03942-9

**Published:** 2023-04-12

**Authors:** Wenqi Qiu, Qian Wu, Kaiwen Zhang, Xiaoli Da, Kairui Tang, Naijun Yuan, Lijuan Deng, Mansi Wu, Ying Zhang, Jiangyan Quan, Qingyu Ma, Xiaojuan Li, Jiaxu Chen

**Affiliations:** 1 School of Traditional Chinese Medicine, Beijing University of Chinese Medicine, Beijing, 100029 China; 2 Formula-pattern Research Center, School of Traditional Chinese Medicine, Jinan University, Guangzhou, 510632 China

**Keywords:** Xiaoyaosan, Depression, Glucose intolerance, LepR-STAT3/PI3K, Chronic unpredictable mild stress

## Abstract

**Background:**

Accumulating evidence has demonstrated that arcuate nucleus (ARC) of the hypothalamus is likely responsible for the close association between chronic stress, depression, and diabetes. Xiaoyaosan (XYS), a Chinese herbal formula, remarkably improves depressive-like behavior and glucose intolerance, but the mechanism remains unclear. Leptin receptor (LepR) regulates energy expenditure and depression by mediating the action of leptin on the ARC. Therefore, we hypothesized that XYS may regulate depressive-like behavior and glucose intolerance via the leptin and its cascade LepR-STAT3/PI3K pathway in the ARC.

**Methods:**

A rat model of depressive-like behavior and susceptibility to glucose intolerance was induced by exposure to chronic unpredictable mild stress (CUMS) for six weeks. XYS (2.224 g/kg) was orally gavaged for six weeks, and fluoxetine (2.0 mg/kg) was administrated to the positive control group. Depressive-like behaviors were assessed using the open field test (OFT), sucrose preference test (SPT) and forced swim test (FST). Fasting blood glucose (FBG) and oral glucose tolerance test (OGTT) were performed to evaluate the effects of XYS on blood glucose. Peripheral leptin and blood lipids were detected using enzyme-linked immunosorbent assay and an automatic biochemical analyzer, respectively. The effects of XYS on the LepR-STAT3/PI3K pathway were detected by quantitative real-time PCR and western blotting.

**Results:**

XYS ameliorated CUMS-induced depressive-like behaviors and elevated blood glucose. XYS improved the food intake but have no significant effects on the body weight. Peripheral leptin and its central receptor were also suppressed by XYS, accompanied by the downregulation of JAK2/STAT3 and PI3K/AKT pathway in the ARC. Additionally, XYS increased AGRP and NPY expression but inhibited POMC in the ARC.

**Conclusions:**

XYS improves depressive-like behaviors and susceptibility to glucose intolerance induced by CUMS, which may be achieved by the downregulation of the LepR-STAT3/PI3K signaling pathway in the ARC.

**Supplementary Information:**

The online version contains supplementary material available at 10.1186/s12906-023-03942-9.

## Introduction

Depression is a common and severe mental disease that has a substantively and negatively impacts on quality of life. According to the World Health Organization, depression accounts for the largest share of the global burden of disease [1], and the overall prevalence of depression has been increasing in recent decades [2]. Notably, depression could increase physical health risk factors, such as obesity, glucose intolerance and cardiovascular disease, of which glucose intolerance is one of the most common comorbidities associated with depression. According to evidence from an epidemiologic survey, it is likely that depression is present in one of every four people with type 2 diabetes mellitus (T2DM) [3]. In turn, approximately 15% of people with diabetes met the criteria for diagnosis of depression in a meta-analysis study [4]. In addition, response to the treatment for depression and diabetes appears to be worse in patients with comorbid depression and diabetes than in those without.

Stress is generally defined as a real or perceived threat to homeostasis or well-being [5]. Therefore, stress could exert numerous effects on behavior and activate physiological stress responses, including the activation of the hypothalamic-pituitary-adrenal (HPA) axis and the sympathetic nervous system. In particular, a hyperactive HPA axis leads to the production of glucocorticoids, which exert numerous effects throughout the brain and body, such as increased liver glucose output, release of fatty acids from white adipose tissue, and reduced insulin secretion [6, 7]. Although the effects of stress are variable and complex, many studies have shown that chronic stress exposure in rodents may impair glucose tolerance and contribute to the development of T2DM [8], which involves repeated immobilization stress [9], swim stress [10], chronic variable stress [11], chronic social stress [12], and chronic unpredictable mild stress (CUMS) [13]. Our previous studies demonstrated that the CUMS induces glucose and lipid metabolism disorders, as well as insulin resistance in rats, which may be closely related to the hypothalamic insulin signaling pathway [14]. Therefore, in this present study, we evaluated the associated mechanism in hypothalamus following CUMS.

The arcuate nucleus (ARC) of the hypothalamus is considered essential for regulating energy homeostasis and depression. The ARC is usually affected by stress through various mechanisms, such as the HPA axis, neural projections in the hypothalamus and neuroendocrine pathways, thus actively involving the development of depression. In contrast, ARC neurons express a high abundance of insulin receptors, which play a vital role in the energy balance by regulating the central and peripheral insulin. More importantly, accumulating evidence has demonstrated that ARC is likely responsible for the close association between chronic stress, depression, and diabetes [15]. This action is dependent on the activation of insulin signaling cascades in ARC, which is mediated by the central LepR-STAT3 pathway. As the most crucial leptin receptors, LepR regulates energy expenditure and food intake by mediating the action of leptin in ARC. Once leptin binds to the LepR in ARC, multiple signaling pathways, including JAK2-dependent and -independent pathways, are activated directly or indirectly. Indeed, the activation of JAK2 and phosphorylation of the JAK2-LepR complex could bind to STAT3 and IRS, respectively, subsequently activating the neuropeptides that regulate energy and insulin signaling. These cascade processes indicate a pivotal role of hypothalamic ARC in energy balance, insulin sensitivity and glucose homeostasis in the whole body.

Depression is associated with glucose intolerance. Thus, a combination of hypoglycemic and antidepressant drugs is widely used as preferred treatment for depressive-like behavior and susceptibility to glucose intolerance. However, several side effects and inadequate responses have been frequently observed [16]. Therefore, to reduce these adverse effects, new insights into complementary and alternative medicines are becoming indispensable for treating depressive-like behavior and susceptibility to glucose intolerance. Xiaoyaosan (XYS) is a classical compound formula designed by *Taiping Huimin Heji Jufang*, a Chinese materia medica officially compiled during the Song Dynasty of China (960–1127 AD) to treat the syndrome of liver Qi stagnation and spleen deficiency in traditional Chinese medicine (TCM). According to the TCM theory, the manifestations of this syndrome mainly include the disorders of emotion and energy metabolism [17]. XYS is composed of eight herbs, including *Radix bupleuri* (root of *Bupleurum chinensis* DC.), *Radix Paeoniae Alba* (root of *Paeonia lactiflora* Pall.), *Radix Angelica sinensis* (root of *Angelica sinensis* (Oliv.) Diels), *Rhizoma Atractylodis* (root and rhizome of *Atractylodes lancea* (Thunb.) DC.), *Poria cocos* (fungus nucleus of *Poria cocos* (Schw.) Wolf), *Liquorice* (root and rhizome of *Glycyrrhiza uralensis* Fisch.), *Mentha haplocalyx* (aboveground portions of *Mentha haplocalyx* Briq.), and *Zingiber officinale* (fresh root and rhizome of *Zingiber officinale* Rosc.). Previous studies have shown that XYS effectively improves depressive behaviors in rodents [18–21], and treats patients with mild depression and its common accompanying symptoms, such as insomnia and fatigue [22]. Moreover, recent network pharmacology focusing on XYS showed that XYS exerted an improved effect on both diabetes and depression involving simultaneous regulation of multiple pathways, such as immune inflammatory reactions, insulin and its receptors (PI3K-AKT), and the cAMP signaling pathway [23]. Recently, we reported that XYS ameliorated the abnormal insulin and blood glucose levels of depressed rats by regulating the PI3K/AKT signaling activity in the liver [24], resulting in decreased blood glucose. These observations suggest that XYS, as an effective antidepressant, may potentially improve glucose intolerance in depression and, more importantly, be a suitable drug for investigating its underlying mechanism to exert multiple therapeutic effects. Taken together, these findings suggest a potential effect of XYS in treating depression and improving blood glucose levels. Although many efforts have been made to explore how XYS to reduce depressive-like behaviors and high blood glucose levels, few studies have focused on investigating the mechanism from the perspective of the ARC, which plays a major role in regulating the energy balance of the body. Fluoxetine, a selective serotonin re-uptake inhibitor antidepressant (SSRI), is an effective agent for improving the depression. Recent studies have demonstrated that fluoxetine effectively reduces depression severity in patients with diabetes with controlled blood glucose [25] and improved insulin sensitivity in patients with obesity and diabetes mellitus [26]. Therefore, we chose fluoxetine as a positive control to compare the effects of XYS in this study. In the current study, we first observed the effects of XYS on depressive-like behavior and the regulation of blood glucose levels. Then, the insulin signaling cascades in ARC were assessed to explore the mechanism by which XYS prevent and treat depressive-like behavior and susceptibility to glucose intolerance, and to further reveal the pathophysiological and pharmacological mechanisms of XYS in depressive-like behavior and susceptibility to glucose intolerance.

## Materials and Methods

### Animals

Male Sprague-Dawley rats were purchased from the Vital River Laboratory Animal Technology Limited Company (Beijing, China). Animals were kept in cages under standard laboratory conditions with a light/dark cycle of 12 h (light phase 6:00–18:00), and food and water were provided *ad* libitum. After 7 days of acclimatization, the rats were subjected to an open field test (OFT) to exclude rats with significant differences. The rats were randomly divided into the control group, the model group, the XYS group and the fluoxetine group. All experiments and animal care were approved by the Ethics Committee of Beijing University of Chinese Medicine (No. BUCM-4-2013101501-4001) and in accordance with the Chinese legislation on the use and care of laboratory animals.

### CUMS procedure

**Fig. 1:**
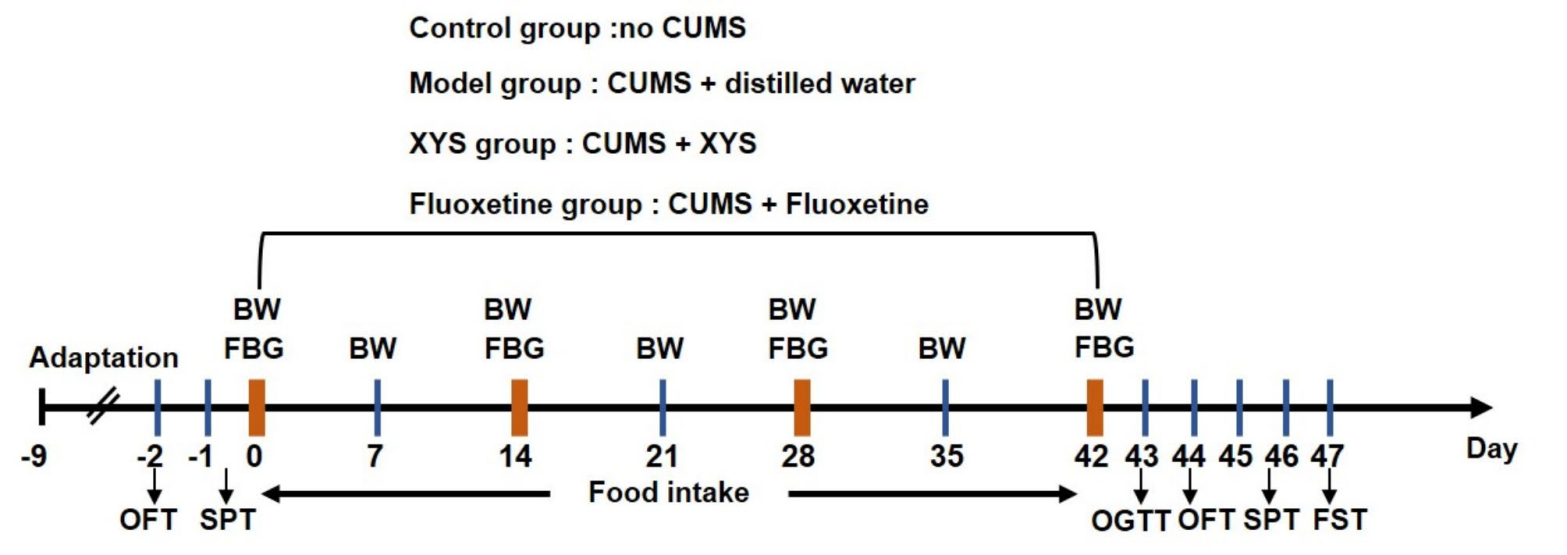
Experiment schedule. After a 7-day adaptation period, rats underwent OFT and SPT to exclude significant different individuals. Rats were exposed to CUMS modeling except for the control group. The daily BW and weekly food intake were monitored, and FBG was monitored every two weeks. After 6 weeks of CUMS modeling, rats in each group underwent OGTT, OFT, SPT, and FST. The rats were sacrificed and tissues were collected to detect the corresponding indicators. BW, body weight; FBG, fasting blood glucose; OGTT, oral glucose tolerance test; OFT, open field test; SPT, sucrose preference test; FST, forced swimming test; CUMS, chronic unpredictable mild stress

The CUMS procedure used in the experiment was performed as described previously [27]. In short, various stressors were randomly administered within 6 weeks, including food and water deprivation for 18 h, swimming in an ice-water mixture for 5 min, heat stress at 45 °C for 5 min, white noise (85 dB) for 5 h, reversed light/dark cycle for 24 h, physical constraint for 3 h, and damp bedding (100 g sawdust bedding with 200 ml water) for 17 h. Except for the control group, rats in other groups were exposed to the above stresses every day for the two continuous days without one same stress. The rats in the control group were kept in a separate room under standard laboratory conditions. During the CUMS procedure, food intake was monitored daily, body weight was measured weekly, fasting blood glucose (FBG) was determined per two weeks, and depressive-like behavioral tests including the open field test (OFT), sucrose preference test (SPT) and forced swim test (FST) and oral glucose tolerance test (OGTT) were performed at the end of the experiment. The experiment schedule is shown as Fig. 1.

### Preparation of XYS

XYS comprises eight Chinese herbs, including *Radix bupleuri* (root of *Bupleurum chinensis* DC.), *Radix Paeoniae Alba* (root of *Paeonia lactiflora* Pall.), *Radix Angelica sinensis* (root of *Angelica sinensis* (Oliv.) Diels), *Rhizoma Atractylodis* (root and rhizome of *Atractylodes lancea* (Thunb.) DC.), *Poria cocos* (fungus nucleus of *Poria cocos* (Schw.) Wolf), *Liquorice* (root and rhizome of *Glycyrrhiza uralensis* Fisch.), *Mentha haplocalyx* (aboveground portions of *Mentha haplocalyx* Briq.), and *Zingiber officinale* (fresh root and rhizome of *Zingiber officinale* Rosc.) with a ratio of 3:3:3:3:3:1.5:1:1. All XYS herbal products were provided by Jiuzhitang Co., Ltd (Changsha, China), and identified by experts at the School of Pharmacy, Beijing University of Chinese Medicine. To ensure the consistency of the compound, XYS was extracted according to the standard methods described in the Pharmacopeia of the People’s Republic of China (China Pharmacopoeia Commission, 2010) as following. First, volatile oil was extracted from the half weight of *Radix bupleuri*, *Radix Angelica sinensis*, and *Mentha haplocalyx* and *Zingiber officinale*. Then, the residue in the volatile oil, *Rhizoma Atractylodis* and *Poria cocos* were mixed and boiled twice for 2 h per time, which was concentrated to a thick paste. *Radix Paeoniae Alba*, the remaining half of *Radix Angelica sinensis* and one-quarter weight of *Liquorice* were crushed to obtain fine powders. The remaining three-quarters of liquor was boiled three times for 2 h each and concentrated into a decoction. Finally, the above volatile oil, thick paste, fine powders, and decoction were mixed uniformly into the decoction and dried and stored at 4 ℃. The quality of XYS was identified by a ultra-performance liquid chromatography (UPLC) coupled with a Waters Acquity UPLC 1-Class system (Waters., Manchester, UK) equipped with a binary solvent delivery manager, as described previously [28]. The major constituents in the mixture included paeoniflorin, kaempferol, quercetin, aloeemodin, saikosaponin A, luteolin, and acacetin, as previously reported [28].

### Drug administration

XYS extract was dissolved in distilled water and administered intragastrically daily at a dose of 2.224 g/kg/day [29], at 0.1 mL/kg body weight. Fluoxetine was dissolved in distilled water and administered intragastrically daily at an effective dose of 2.0 mg/kg/day at 0.1 mL/kg body weight according to a previous experiment [30]. The model group was intragastrically administered the same volume of distilled water daily, according to the body weight.

### Behavioral tests

#### OFT

 This test was performed as described previously [20]. The open field consisted of a square arena (100 × 100 cm) with 25 squares on the ground and a 20 cm wall around it. A camera was attached to a computer above the arena. The rats were individually placed in the center of the arena and allowed free exploration for 5 min. The rat behaviors in the arena were recorded by Observer 5.0 software (Noldus, Wageningen, Netherlands) and analyzed by EthoVision 14.0 software (Noldus, Netherlands), including the total distance moved and time spent in the central area.

#### SPT

All rats were trained for 72 h firstly. During the first 24 h, the rats in each cage were given two bottles of 1% sucrose solution for adaptation. During the second 24 h, the rats were given one bottle of 1% sucrose solution and one bottle of pure water. Two bottles were removed, and the rats were fasted for water and food for 24 h. Each rat was then individually separated into cages and allowed 1 h of free access to a bottle of 1% sucrose solution and a bottle of pure water. Each bottle was weighed, and the sucrose preference rate was calculated as follows: sucrose solution consumption / [pure water consumption + sucrose solution consumption] × 100%.

#### FST

The test was included a 15-min pre-test and a 5-min swim test after 24 h. First, each rat was placed individually in a plastic tank with a water depth of 30 cm and allowed to swim for 15 min. After 24 h, each rat was placed in a plastic tank and forced to swim for 5 min. The rat behaviors in the arena were recorded using Observer 5.0 software (Noldus, Netherlands), and the immobility time was analyzed using EthoVision software (version 14.0 Noldus, Netherlands).

### FBG, triglyceride, total cholesterol, high- and low-density lipoprotein, body weight and food intake

Blood samples were collected from the tail after fasting for 14 h. FBG was tested using a blood glucose monitoring system (Johnson). Abdominal aortic blood samples were analyzed with a fully automatic biochemical analyzer (Beckman, USA) to determine the triglyceride (TG), total cholesterol (TC), and high- and low-density lipoprotein (HDL and LDL, respectively) levels.

Body weight during the adaptation period was measured before the experiment as a baseline and subsequently weekly throughout the study. Food intake was monitored over 24 h and determined by subtracting the amount of remaining food, including the amount of spilled food on the bottom of the cage, from their respective amount on the previous day.

### OGTT

The OGTT was measured after an 18-hour overnight fast. A solution of glucose (2 g/kg) was administered by oral gavage. Blood glucose concentrations from the tail tip were determined at 0, 30, 60, 90 and 120 min after the administration of glucose solution infusion using a blood glucose monitoring system (Johnson).

### Enzyme-linked immunosorbent assay analysis

An enzyme-linked immunosorbent assay (ELISA) kit (RayBiotech, USA) was used to quantify serum leptin levels in rats. Standards and samples were added into each well, and leptin in the sample was combined with the wells using an immobilized antibody. After washing four times, biotinylated anti-rat leptin antibody was added to the wells. Horseradish peroxidase (HRP)-conjugated streptavidin was then added to the wells after washing away the unbound biotinylated antibody. The wells then washed again with TMB substrate solution, and the color developed was consistent with the amount of bound leptin. Color intensity was measured at 450 nm after adding stop solution.

### Real-time quantitative PCR analysis

Total RNA was isolated from ARC tissues using TRIzol reagent (Life Technologies, USA) according to the instructions and stored at -80 ℃. The total RNA was reverse transcribed into cDNA using PrimeScript™ RT Master Mix (Takara, Cat. RR036A) on a Mastercycler® nexus gradien (Eppendorf, Germany). cDNA was amplified using the TB Green™ Advantage® qPCR Premix kit (Takara, Cat. 639,676) on a CFX96 Real-time PCR System (Bio-Rad, USA). The total amplification volume was 25 µL, and the added volumes of TB Green *Premix Ex Taq* II, forward primer (10 µM), reverse primer (10 µM), cDNA (98 ng/µL) and RNase-free dH2O were 12.5, 1, 1, 2 and 8.5 µL respectively. The amplification condition was as follows: 5 °C for 30 s, and 40 cycles of 95 °C for 5 s, and 60 °C for 30 s. The amplification reaction was performed in triplicate. Relative quantification of gene expression was normalized to GAPDH gene expression using the 2^–ΔΔCt^ method. The primer sequences used are listed in Table 1.

**Table 1:** Sequence of oligonucleotides used for RT-qPCR

Gene	Forward primer	Reverse primer	Annealing temperatures (°C)
LepR	CCGAGAAGATCCCCGAGACA	ACTTCCCTCGAGGTCTGGTG	60
JAK2	GTGCGTGCGAGCGAAGATCC	ACTGCTGAATGAACCTGCGGAATC	60
STAT3	CCAGTCGTGGTGATCTCCAACATC	CAGGTTCCAATCGGAGGCTTAGTG	60
POMC	AGGCGTGCGGAGGAAGAGAC	GACTCGTTCTCGGCGACATTGG	60
AGRP	GCAGCAGACCGAGCAGAAGATG	GCACAGGTCGCAGCAAGGTAC	60
NPY	TGCTCGTGTGTTTGGGCATT	GATGTAGTGTCGCAGAGCGG	60
GAPDH	CCATTCTTCCACCTTTGAT	TGGTCCAGGGTTTCTTACT	58
GLUT4	AGCCAGCCTACGCCACCATAG	CAGCAGAGCCACCGTCATCAAG	60
IRS-1	AGCAACAGCAGCAGCAGTCTTC	ACTCTTCCGAGCCAGTCTCTTCTC	60
PI3K	AACTCGCCTCATAGCAGAGCAATG	TGGCACGCAGTCATGGTTGATC	60
AKT	GGCAGGAGGAGGAGACGATGG	TTCATGGTCACACGGTGCTTGG	60

### Western blotting (WB) analysis

ARC tissues were homogenized to extract the total protein using RIPA lysis buffer (Beyotime, Cat. P0013B) containing protease inhibitor (Boster, Cat. AR1182) and phosphatase inhibitor (Boster, Cat. AR1183). Protein (30 µg) was subjected to 10% sodium dodecyl-sulfate-polyacrylamide gel electrophoresis (SDS-PAGE), followed by transferring into polyvinylidene difluoride (PVDF) using a standard semi-dry transfer system (Bio-Rad, USA). The transferred PVDF membranes were blocked with 5% non-fat milk for 1 h, cutted according to respective molecular weight and then incubated with the primary antibody (LEPR, Abcam, Cat. ab5593; p-JAK2 (Tyr1007), Cell Signaling Technology, Cat.#3771S; JAK2, Cell Signaling Technology, Cat.#3230; p-STAT3 (Tyr795), Cell Signaling Technology, Cat.#4113; STAT3, Cell Signaling Technology, Cat.#4904; p-IRS-1 (Ser307), Cell Signaling Technology, Cat. #2381; IRS-1, Cell Signaling Technology, Cat. #2390; p-PI3K p85(Tyr458)/p55(Tyr199), Cell Signaling Technology, Cat. #4228; PI3K, Cell Signaling Technology, Cat. #4249; p-AKT (S473), Abcam, Cat. #5174; AKT, Proteintech, Cat. 60203-2-Ig; GLUT4, Cell Signaling Technology, Cat. #2213; β-actin, Proteintech, Cat. 66009-1-Ig; GADPH, Cell Signaling Technology, Cat. #5174) overnight at 4 °C. The membranes were then washed and incubated with HRP‐conjugated secondary antibodies at room temperature for 1 h. Chemiluminescence was detected using an enhanced chemiluminescence reagent (Bio-rad, USA) on a ChemiDocTM Imaging System (Bio-rad, USA). Image J (National Institutes of Health, Bethesda, MD) software was used to quantify the density of specific bands.

### Statistical analysis

All data were analyzed by SPSS 20.0 software (IBM, Armonk, NY) and expressed as mean ± Standard Deviation ( $\bar{\text{x}}$ ± S.D.). Data of food intake, body weight, FBG and OGTT data were analyzed using a repeated measure analysis of variance (ANOVA) to determine significant differences between time and stress. Other data were analyzed using a one-way ANOVA. Statistical significance was assessed using one-way analysis of variance (ANOVA) followed by *Bonferroni’s post hoc* test for multiple comparisons. P value < 0.05 was considered as a statistically significant difference. All figures were plotted with GraphPad Prism 7.0 Software (GraphPad Software, San Diego, CA).

## Results

### Effects of XYS on depressive behaviors of CUMS rats

**Fig. 2:**
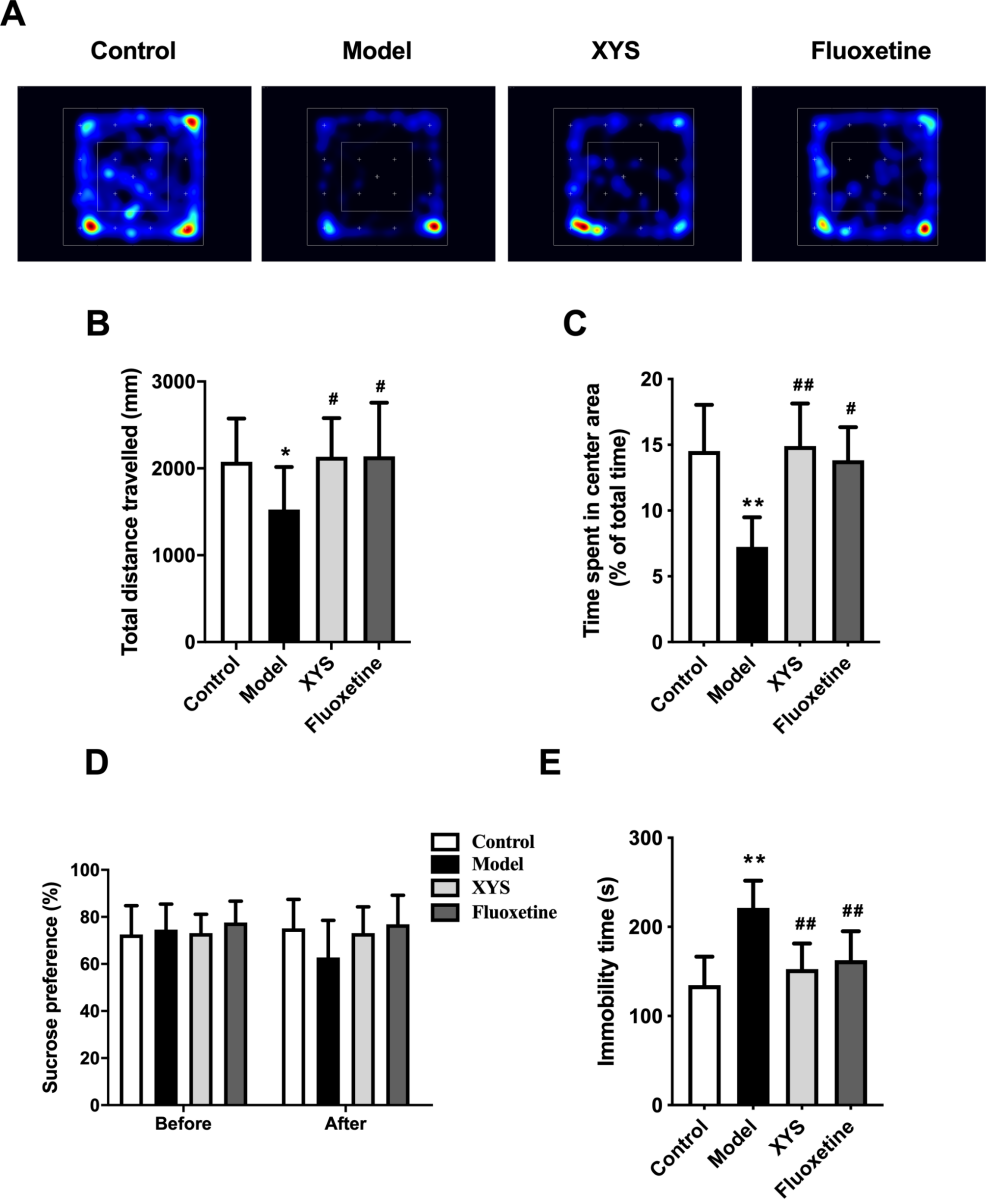
Effects of XYS on depressive-like behaviors of rats **(A)** Behavioral track heatmap of rats in the OFT. **(B)** Total distance traveled in the OFT. **(C)** Time spent in the center of the OFT. **(D)** Sucrose preference in the SPT before and after establishing CUMS modeling. **(E)** Immobility time in the FST. Data are presented as mean and standard deviation. n = 10, 10–14, 11–14 per group in OFT, SPT and FST, respectively; *P < 0.05, **P < 0.01, vs. control group; ^#^P < 0.05, ^##^P < 0.01, vs. model group. XYS, Xiaoyaosan; OFT, open field test; SPT, sucrose preference test; FST, forced swim test; CUMS, chronic unpredictable mild stress

The SPT, OFT and FST can reflect the depressive behaviors of rats from different perspectives. The OFT assesses the locomotor activity in rats, and a decrease in locomotor activity indicates anxiety-like behavior, which is associated with depression [31]. The behavioral track heat map of the rats is shown in Fig. 2A. The total distance traveled and the time spent in the center area were decreased, indicating that the locomotor activity of rats in the OFT was reduced, whereas XYS reversed these changes (P > 0.05 or P < 0.01, Fig. 2B and C). Rodents like to eat sweets food, and reduced sucrose consumption in the SPF can reflect a lack of pleasure, which is a manifestation of depression [32]. As shown in Fig. 2D, the rats in each group were all at the baseline level before modeling, whereas the rats in the model group had a reduced preference for sucrose after 6 weeks of CUMS modeling, with no statistical difference (P > 0.05, Fig. 2D). The particular form of immobility observed in the FST is termed behavioral despair, which is also a manifestation of depression [33]. As shown in Fig. 2E, the immobility time in the model group was increased, whereas XYS reversed this effect (P < 0.01, Fig. 2E).

### Effects of XYS on glucose intolerance, leptin, body weight and food intake of CUMS rats

**Fig. 3:**
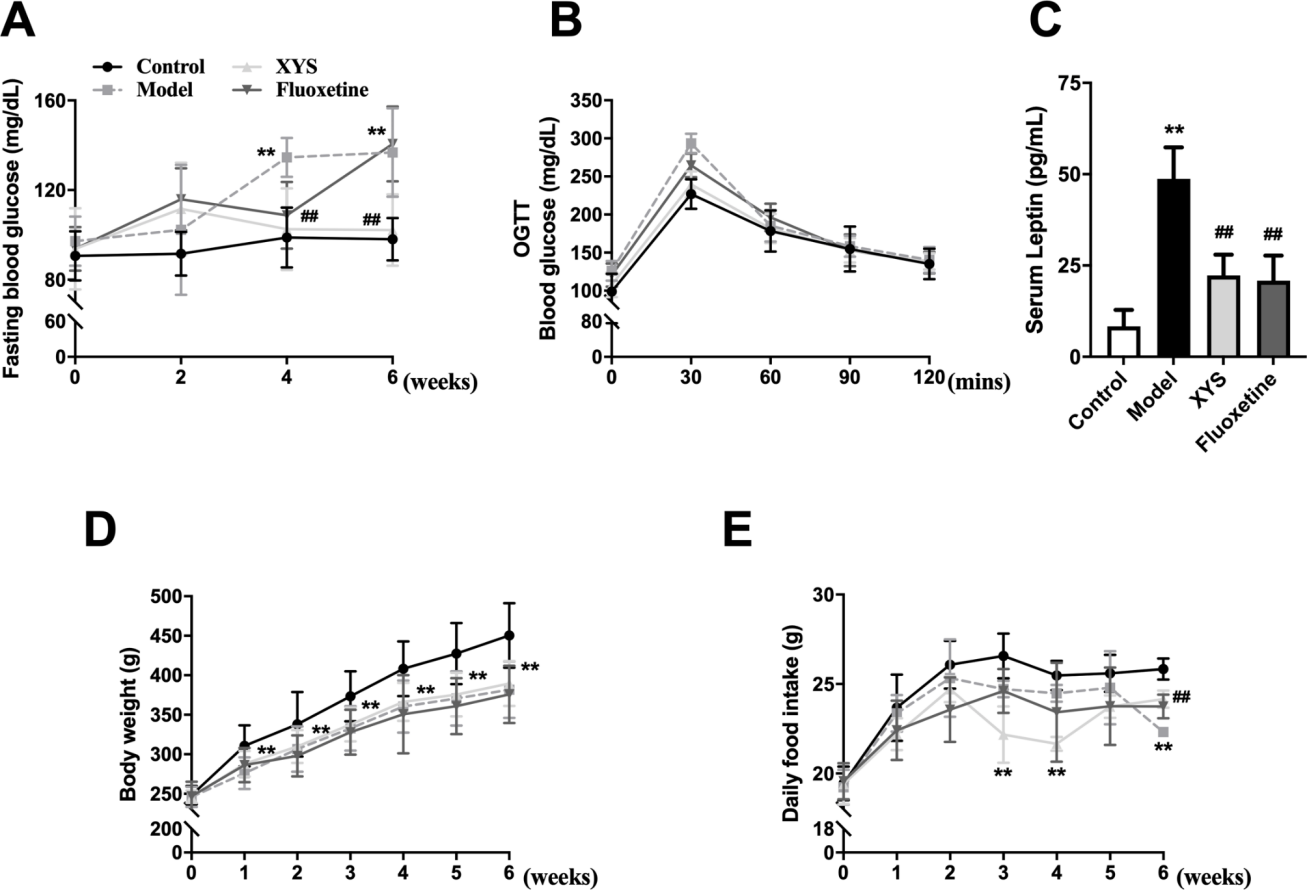
Effects of XYS on glucose intolerance in CUMS rats. **(A)** FBG. **(B)** OGTT. **(C)** Serum leptin. **(D)** Body weight. **(E)** Daily food intake. Data are presented as mean and standard deviation, n = 11–14 in A, B, D and E per group; n = 10 in C per group; *P < 0.05, **P < 0.01, vs. control group; ^#^P < 0.05, ^##^P < 0.01, vs. model group. XYS, Xiaoyaosan; FBG, fasting blood glucose; OGTT, oral glucose tolerance test; CUMS, chronic unpredictable mild stress

With increasing exposure to CUMS, FBG increased gradually, with a statistical difference at week 4 and 6 (P < 0.01, Fig. 3A). XYS lowered FGB levels to the almost normal levels (P < 0.01, Fig. 3A). The blood glucose level in the model group was higher than that in the control group, and XYS lowered this change at 30 min; however, this difference was no significance (P > 0.05, Fig. 3B). According to these data, we conducted Pearson correlation analysis for the correlation between immobility time in FST and the FBG level of rats at 6 weeks. The results showed that there was a significant correlation between the two groups of data (r^2^ = 0.2556, P = 0.0012), as shown in the Supplement Fig. 1. In adipose tissue, leptin controls energy balance and food intake, in part by promoting or inhibiting hypothalamic neurons in the ARC [34]. The leptin level of the model group increased, which was consistent with the weight loss of the model group, and XYS reversed this change (P < 0.01, Fig. 3C). Body weight and food intake were monitored weekly. Body weight gradually increased over time, with a significant decrease in the model group compared with the control group (P < 0.01, Fig. 3D). As shown in Fig. 3E, the food intake of the CUMS group was lower than that of the control group (P < 0.01). But, XYS could obviously reverse the food intake (P < 0.01) but have no significant effects on the body weight (P > 0.05), as shown in Fig. 3D and E.

### Effects of XYS on peripheral TG, TC, HDL, LDL in CUMS rats

Indicators of lipid metabolism are shown in Table 2. Compared to the control group, TG and TC levels in the model group decreased, but not significantly (P > 0.05). There was no significant difference in HDL between groups. Compared to the control group, LDL also decreased significantly in the model group (P < 0.05), whereas XYS lowered the LDL level compared to the model group (P < 0.01). The results on lipid metabolism are interesting and require further study.

**Table 2:** Effects of XYS on peripheral TG, TC, HDL, and LDL in CUMS rats

Group	TG (mmol/L)	TC (mmol/L)	HDL (mmol/L)	LDL (mmol/L)
Control	0.396 ± 0.141	1.348 ± 0.293	0.307 ± 0.038	0.273 ± 0.092
Model	0.317 ± 0.050	1.189 ± 0.263	0.303 ± 0.080	0.212 ± 0.036^*^
XYS	0.386 ± 0.113	1.375 ± 0.164	0.338 ± 0.052	0.202 ± 0.035^**##**^
Fluoxetine	0.360 ± 0.068	1.164 ± 0.154	0.301 ± 0.065	0.226 ± 0.048

Data are presented as mean and SD. n = 11–14 per group; *P < 0.05, **P < 0.01, vs. control group; ^#^P < 0.05, ^##^P < 0.01, vs. model group. *TG* triglyceride; *TC* total cholesterol; *HDL* high-density lipoprotein; *LDL* low-density lipoprotein.

### Effects of XYS on GULT4 in hypothalamic ARC of CUMS rats

**Fig. 4:**
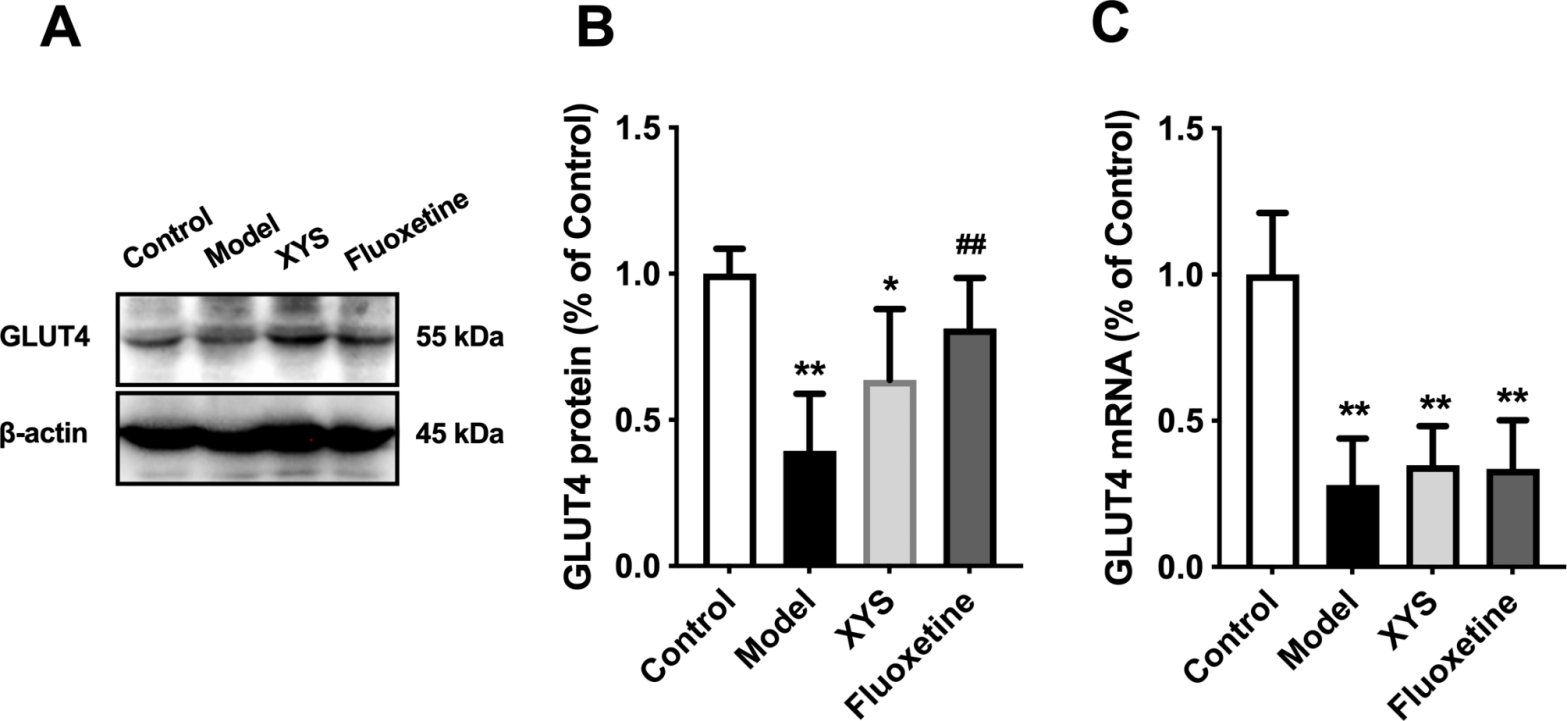
Effects of XYS on GLUT4 in ARC of CUMS rats. **(A)** Representative immunoblots of GLUT4. **(B)** Quantitative protein data of GLUT4. **(C)** mRNA expression of GLUT4. Data are presented as mean and standard deviation. n = 5 per group; *P < 0.05, **P < 0.01, vs. control group; ^#^P < 0.05, ^##^P < 0.01, vs. model group. XYS, Xiaoyaosan; CUMS, chronic unpredictable mild stress

The protein and mRNA expression levels of GLUT4 are shown in Fig. 4. GLUT4 protein decreased in the CUMS group (P < 0.01, Fig. 4A and B), and XYS and fluoxetine reversed this change, but the effect of XYS was not significant (P > 0.05). CUMS resulted in a decrease in GLUT4 mRNA expression (P < 0.01, Fig. 4C); however, drug intervention did not reverse this change. This result is opposite to that of protein expression, suggesting that GLUT4 protein expression is related to other protein production processes, such as post-translational modification.

### Effects of XYS on LepR-STAT3 pathway in ARC of CUMS rats

**Fig. 5:**
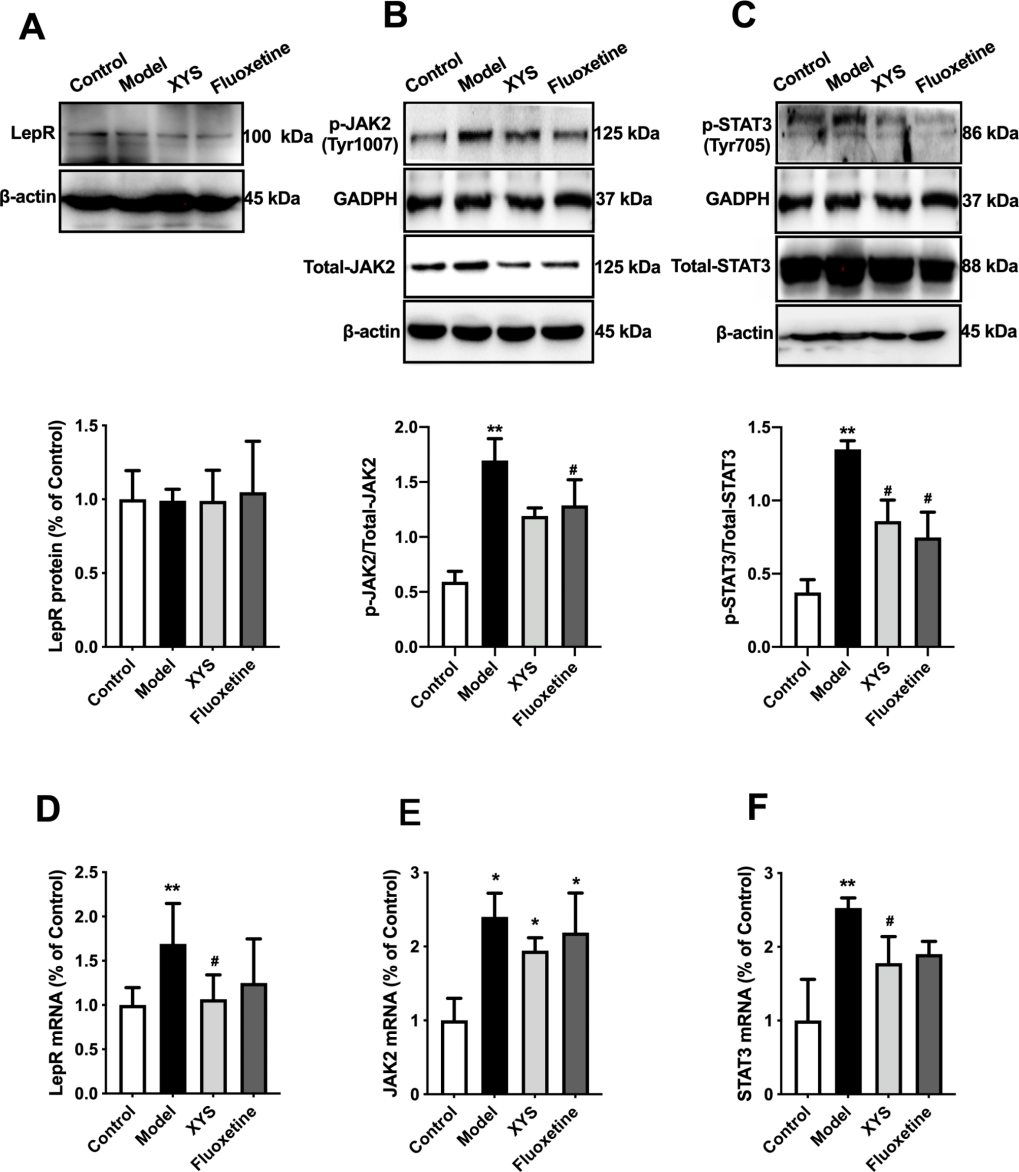
Effects of XYS on LepR-STAT3 pathway in ARC of CUMS rats. **(A)** Representative images for immunoblots and quantitative protein data of LepR. **(B)** Representative images for immunoblots of p-JAK2 and JAK and quantitative protein ratio of p-JAK2/JAK. **(C)** Representative images for immunoblots of p-STAT3 and STAT3 and quantitative protein ratio of p-STAT3/STAT3. **(D)** PCR results of LEPR. **(E)** PCR results of JAK2. **(F)** PCR results of STAT3. Data are presented as mean and standard deviation. n = 4 per group; *P < 0.05, **P < 0.01, vs. control group; ^#^P < 0.05, ^##^P < 0.01, vs. model group. XYS, Xiaoyaosan; CUMS, chronic unpredictable mild stress

Protein and mRNA expression levels of the LepR-STAT3 pathway are shown in Fig. 5. LepR protein did not differ between groups, whereas the ratio of p-JAK2/JAK2 and p-STAT3/STAT3 increased in the model group (both P < 0.01; Fig. 5A and B C), and XYS decreased the ratio of p-STAT3/STAT3 (P < 0.05, Fig. 5A and B C). LEPR, JAK2, and STAT3 mRNA levels in the model group increased to varying degrees (P < 0.01 and < 0.05 respectively; Fig. 5D and E F), and XYS reversed the increase in mRNA levels of LEPR and STAT3 (P < 0.05, Fig. 5D and E F).

### Effects of XYS on PI3K signaling in ARC of CUMS rats

**Fig. 6:**
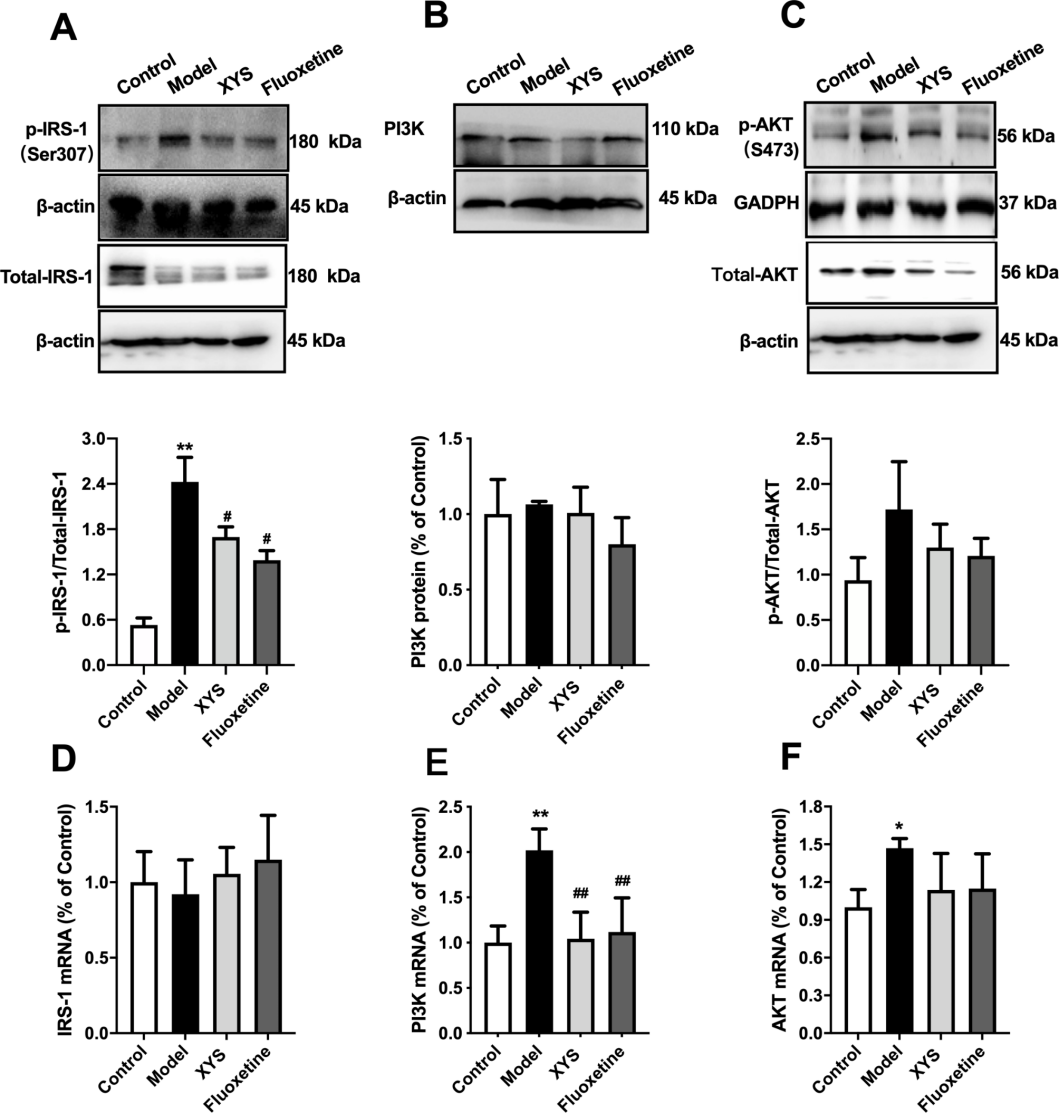
Effects of XYS on PI3K signaling pathway in ARC of rats. **(A)** Representative images for immunoblots of p-IRS-1 and IRS-1 and quantitative protein ratio of p-IRS-1/IRS-1. **(B)** Representative images for immunoblots and quantitative protein data of PI3K. **(C)** Representative images for immunoblots of p-AKT and AKT and quantitative protein ratio of p-AKT/AKT. **(D)** PCR results of IRS-1. **(E)** PCR results of PI3K. **(F)** PCR results of AKT. Data are presented as mean and standard deviation. n = 4 per group; *P < 0.05, **P < 0.01, vs. control group; ^#^P < 0.05, ^##^P < 0.01, vs. model group. XYS, Xiaoyaosan

The protein and mRNA expression levels of the PI3K signaling pathway are shown in Fig. 6. The LepR-STAT3 and PI3K signaling pathways were partially activated, and were repressed with XYS. CUMS modeling increased the ratio of p-IRS-1/IRS-1 (P < 0.01, Fig. 6A) and the mRNA expression of PI3K and AKT (P < 0.01 and < 0.05, respectively; Fig. 6E F), while the ratio of p-IRS-1/IRS-1 and the mRNA expression of PI3K could be reversed by XYS (P < 0.01 and < 0.05, respectively; Fig. 6A and E).

### Effects of XYS on POMC, AGRP and NPY mRNA in hypothalamic ARC of CUMS rats

**Fig. 7:**
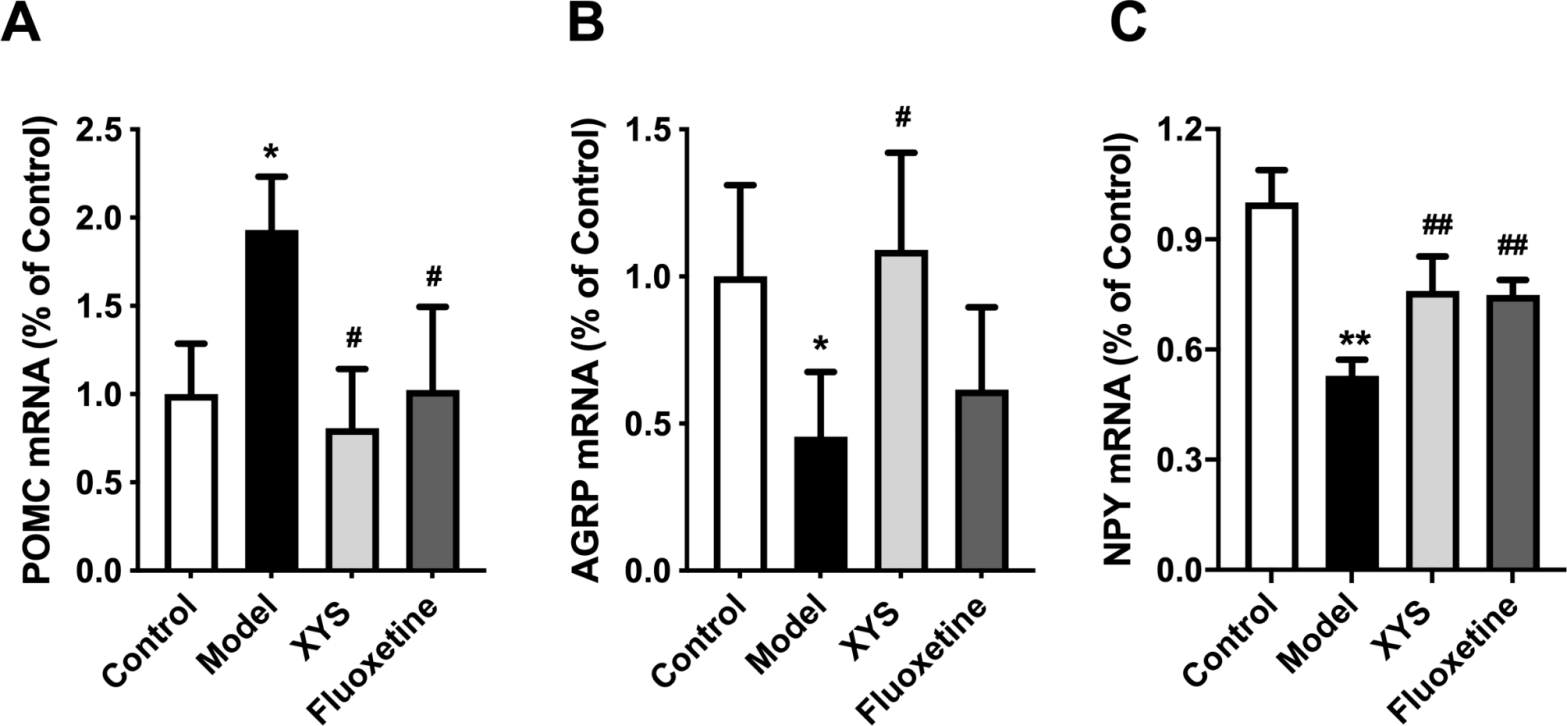
Effects of XYS on POMC, AGRP, and NPY mRNA in ARC of CUMS rats. **(A)** PCR result of POMC. **(B)** PCR result of AGRP. **(C)** PCR result of NPY. Data are presented as mean and standard deviation. n = 5 per group; *P < 0.05, **P < 0.01, vs. control group; ^#^P < 0.05, ^##^P < 0.01, vs. model group. XYS, Xiaoyaosan; CUMS, chronic unpredictable mild stress

POMC, AGRP, and NPY mRNA levels, which are downstream of STAT3 and important molecules regulating appetite, were detected. CUMS modeling increased POMC expression (P < 0.05, Fig. 7A) and decreased AGRP and NPY expression (P < 0.05, Fig. 7B and P < 0.01, Fig. 7C; respectively), consistent with the results of food intake. XYS reversed these changes (P < 0.05, Fig. 7A and B; P < 0.01, Fig. 7C).

## Discussion

**Fig. 8:**
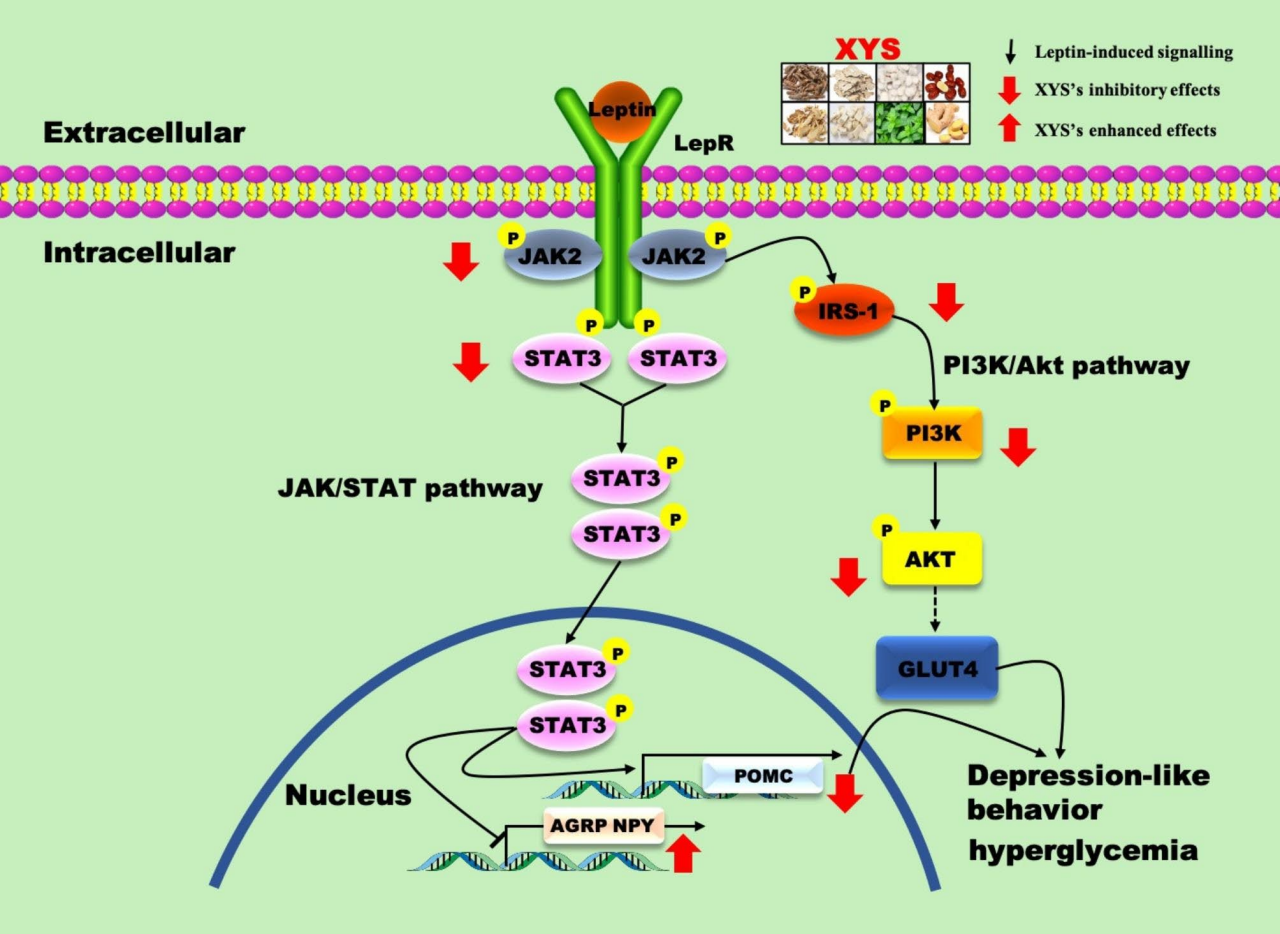
Potential mechanism of Xiaoyaosan underling its improving effects on depressive-like behavior and susceptibility to glucose intolerance of rats via LepR-STAT3/PI3K pathway in the ARC

The purpose of the present study was to investigate the effects of XYS on depressive-like behavior and susceptibility to glucose intolerance and to explore the potential mechanisms underlying these effects. In this study, we confirmed that XYS exerts a significant effect on depressive-like behavior and susceptibility to glucose intolerance by improving depressive-like behaviors, peripheral glucoses. Furthermore, it was found that the mechanism underlying its ameliorative effects on depressive-like behavior and susceptibility to glucose intolerance may be associated with inhibitory effects on the LepR-STAT3/PI3K pathway, thus enhancing AGRP and NPY expression and inhibiting POMC transcription, as shown in Fig. 8.

Clinical studies have shown that patients with depression are more prone to develop glucose metabolism disorder [35–37], and preclinical studies found that the CUMS model showed depressive-like behavior and glucose intolerance [15, 38], suggesting that the CUMS model is suitable for investigating the effect of drugs on depressive-like behavior and susceptibility to glucose intolerance. In this study, the decreased locomotor activity in the OFT and the increased immobility time in the FST confirmed that CUMS is successful in simulating depressive-like behavior, and the increased FBG proves that this model can be used to investigate glucose metabolism problems, which is consistent with previous reports [39–43]. There were no significant changes in the blood lipid levels observed in our study, which may be related to the different CUMS intervention times. In this study, XYS significantly improved depressive-like behavior and susceptibility to glucose intolerance induced by CUMS, as evidenced by changes in the OFT and FST. The therapeutic effects of XYS were consistent with those of clinical reports and our previous study.

The ARC is a prominent neural nucleus involved in regulating diverse biological processes, including appetite control, maintenance of body weight, response to insulin actions and coping with stress. GLUT4 is a major glucose receptor, and its translocation plays a crucial role in glucose metabolism [44]. Once GLUT4 in skeletal muscle or adipose tissue is disrupted, glucose fails to enter the cell, contributing to glucose tolerance and impaired insulin resistance. Recently, a selective knockout of GLUT4 in the brains of mice showed glucose intolerance, hepatic insulin resistance, and decreased glucose uptake in the brain, suggesting a critical role of brain GLUT4 in whole-body glucose homeostasis [45]. In the present study, GLUT4 expression was reduced in the ARC, which is consistent with the reduced GLUT4 expression in the hypothalamus of CUMS-induced depressed mice in our previous study [46]. In fact, extensive research has focused on the role of brain GLUT in stress-induced glucose metabolism disorders, in which GLUT1 and GLUT4 are the main glucose transporters involved in stress affecting systemic glucose homeostasis [47, 48]. Therefore, our findings demonstrate that GLUT4 in the ARC actively participates in the pathogenesis of depressive-like behavior and susceptibility to glucose intolerance. But, in current study, we found that the disorder of GLUT4 in ARC was not significantly reversed by the treatment of XYS, suggesting the effects of XYS on the anti-depression and glucose control may be rarely attributed to the regulation of GLUT4 in the ARC.

LepR is the most critical leptin receptor in the hypothalamus, and is activated by peripheral leptin. Activated LepR in hypothalamic neurons can regulate feeding, energy expenditure, and glucose homeostasis by binding several putative JAK and STAT sites, and consequently influences energy balance in both animals and humans [49–51]. Once leptin binds to LepR in the hypothalamus, downstream cascading signaling is triggered by phosphorylating JAKs and STATs and sequentially inducing the translocation of STAT into the nucleus to regulate the transcription of genes involved in energy homeostasis, such as POMC, NPY, and AGRP [52]. Importantly, in mammals, activated JAK2, which induces the phosphorylation of STAT3, plays crucial roles in the regulation of energy homeostasis by LepR [53, 54]. Meanwhile, there is a negative feedback mechanism in leptin-induced STAT3 signaling through the induction of SOCS3 [55]. Notably, activated LepR also induces phosphorylation of IRS members by binding to the JAK2 site. The activated IRS then recruits and activates PI3K to sequentially activate AKT, consequently stimulating the transcription of energy metabolism genes, including POMC, NPY, and AGRP [56]. Simultaneously, the roles of LepR in the ARC in energy metabolism, including glucose homeostasis, are mainly involved in the LepR-STAT3 signaling pathway and its linked PI3K pathway. Many studies have shown that XYS can reduce leptin in the serum and hypothalamus and reduce LepR in the ARC in animal models [57, 58]. XYS can downregulate the levels of JAK2, phosphorylated -JAK2 and STAT3 in the hippocampus of rats with anxiety behaviors [20]. Network pharmacology shows that STAT3 is one of antidepressant targets of XYS [59]. Additional evidence indicates that XYS exerts its antidepressant effect by modulating the PI3K/AKT signaling pathway in peripheral organ or the brain [24, 60]. As shown in the present study, rats exposed to stress showed activation of the LepR-STAT3 signaling pathway in the ARC, as evidenced by upregulation of JAK2 and STAT3, suggesting that the LepR-STAT3 signaling pathway in the ARC is involved in the pathology of depression induced by CUMS and susceptibility to glucose intolerance. Additionally, our findings showed an activating effect on the PI3K signaling pathway. These changes led to changes in POMC, NPY, and AGRP, which control appetite. However, these changes in the LepR-STAT3 signaling pathway and its related PI3K pathway were partly reversed after treatment with XYS, which exerted inhibitory effects on the LepR-STAT3 signaling pathway and simultaneously promoted the induction of the PI3K pathway. Additionally, XYS reversed the changes in POMC, NPY, and AGRP, which is consistent with previous reports that XYS could regulate the expression of POMC and NPY in the hypothalamus of depressed rats [57, 58, 61]. Interesting, we found that although XYS exerted effects on the peripheral Leptin and POMC and NPY in ARC, only the feeding was significantly improved by the treatment of XYS, suggesting may involve other orexin peptides and molecular pathways. Above all, the effects of XYS may be partially attributed to inhibition of the LepR-STAT3/PI3K pathway. Thus, the mechanism underlying antidepressant and anti-glucose intolerance were partly explained. However, the regulation of stress-induced central metabolism disorders in peripheral glucose intolerance is complex. It is not clear yet how the dysfunction of the LepR-STAT3/PI3K pathway in ARC influences the peripheral glucometabolic tissues, including fat, skeletal muscle, and liver, and the underlying mechanism of XYS. In addition, XYS is composed of eight herbs with multiple chemical composition, such as paeoniflorin, kaempferol, quercetin, aloeemodin, saikosaponin A, luteolin, acacetin, etc. The specific compounds in XYS that play vital roles in ameliorating depressive-like behavior and susceptibility to glucose intolerance are still unknown. Thus, future studies could fruitfully explore this issue further.

## Conclusion

This study demonstrated that XYS could ameliorate CUMS induced depressive-like behavior and susceptibility to glucose intolerance, which may be associated with regulating the LepR-STAT3/PI3K pathway in the ARC of the hypothalamus in rats. These findings not only help us understand the effects and underlying mechanism of XYS on depressive-like behavior and susceptibility to glucose intolerance, but also provide new evidence for the clinical use of XYS as a treatment for depressive-like behavior and susceptibility to glucose intolerance.

## Electronic supplementary material

Below is the link to the electronic supplementary material.

**Additional file 1.** Supplementary figures

## Data Availability

The datasets used and/or analyzed during the current study are available from the corresponding author upon reasonable request. We clarify the data used in this study can be deposited publically.

## Abbreviations

ANOVA: Analysis of variance
ARC: Arcuate nucleus
CUMS: Chronic unpredictable mild stress
ELISA: Enzyme-linked immunosorbent assay
FBG: Fasting blood glucose
FST: Forced swimming test
HDL: High-density lipoprotein
HPA: Hypothalamic-pituitary-adrenal
HRP: Horseradish peroxidase
LDL: Low-density lipoprotein
OFT: Open field test
OGTT: Oral glucose tolerance test
PVDF: Polyvinylidene fluoride
RT-qPCR: Quantitative real-time polymerase chain reaction
SPT: Sucrose preference test
T2DM: Type 2 diabetes mellitus
TC: Total cholesterol
TCM: Traditional Chinese medicine
TG: Triglyceride
UPLC: Ultra-performance liquid chromatography
WB: Western blotting
XYS: Xiaoyaosan
