# Supplementary material for: Xiaoyaosan ameliorates depressive-like behavior and susceptibility to glucose intolerance in rat: involvement of LepR-STAT3/PI3K pathway in hypothalamic arcuate nucleus
Source: BMC Complement Med Ther. 2023 Apr 12;23:116. doi: 10.1186/s12906-023-03942-9 (PMC10091664; doi:10.1186/s12906-023-03942-9)
Supplement: Supplementary file 1 — Additional file 1. Supplementary figures [file 12906_2023_3942_MOESM1_ESM.pdf]

Supplemental Files for:

Xiaoyaosan ameliorates depressive-like behavior and susceptibility to glucose intolerance in rat: involvement of LepR-STAT3/PI3K pathway in hypothalamic arcuate nucleus

Wenqi Qiu[1+], Qian Wu[1+], Kaiwen Zhang[1+], Xiaoli Da[2], Kairui Tang[2], Naijun Yuan[2], Lijuan Deng[2], Mansi Wu[2], Ying Zhang[2], Jiangyan Quan[2], Qingyu Ma[2], Xiaojuan Li[2*], Jiaxu Chen[1,2*]

Institutional address: [1]School of Traditional Chinese Medicine, Beijing University of Chinese Medicine, Beijing 100029, China. [2]Formula-pattern Research Center, School of Traditional Chinese Medicine, Jinan University, Guangzhou 510632, China.

[+] Wenqi Qiu, Qian Wu and Kaiwen Zhang contributed equally to this work.

[*]Correspondence should be addressed to Jiaxu Chen and Xiaojuan Li; E-mail address: chenjiaxu@hotmail.com, lixiaojuan@jnu.edu.cn.

Supplement figure 1

We conducted Pearson correlation analysis for the correlation between immobility time in FST and the fasting blood glucose level of rats at 6 weeks. The results showed that there was a significant correlation among the four groups of data ($r^2 = 0.2556$, $P = 0.0012$), as shown in the following figure.

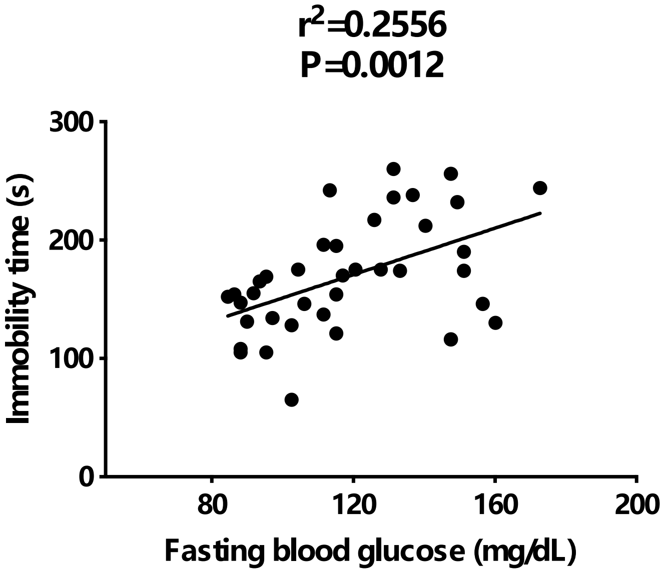

Figure S1. Pearson correlation analysis for the correlation between immobility time in FST and the fasting blood glucose level of rats at 6 weeks. FST: Forced swimming test.

Supplement figure 2

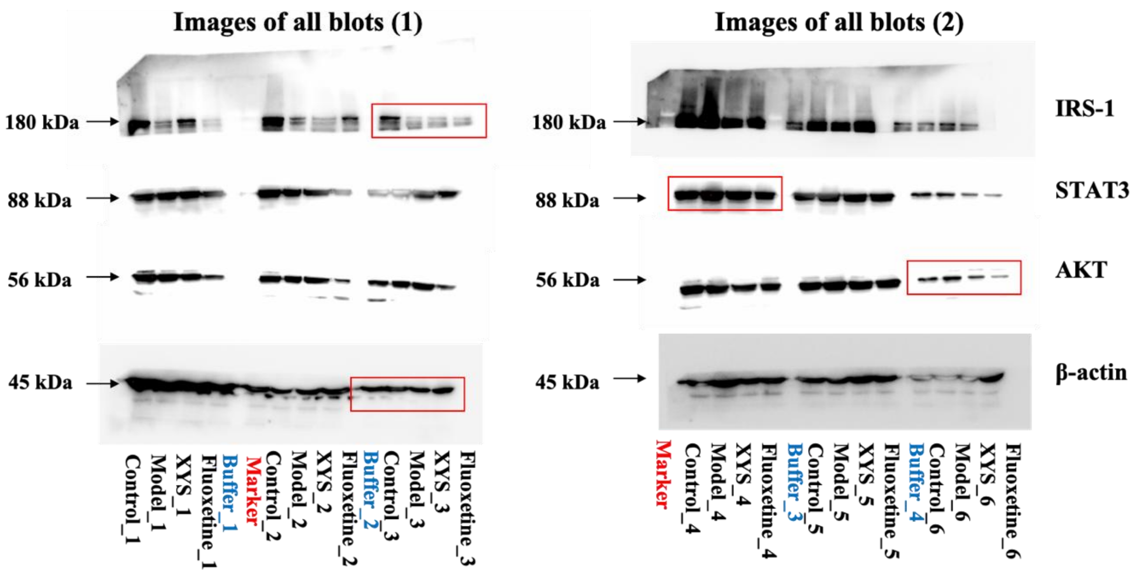

Figure S2. Original images of all blots of IRS-1, STAT3, AKT and β-actin. The images of all blots for all replicates performed for IRS-1 (180 kDa), STAT3 (88 kDa), AKT (56 kDa) and β-actin (45 kDa). Figure S2 corresponds to the Western blot analysis shown in Figure 5C, 6A and 6C of main manuscript. n=6 per group. The red boxes represent the regions of the original blots used in main figures of manuscript.

Supplement figure 3

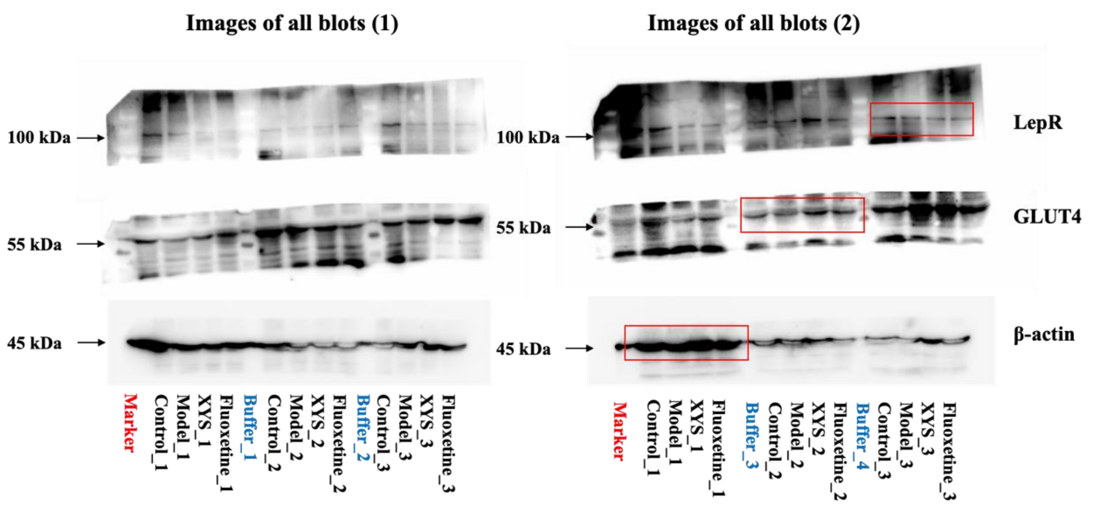

Figure S3. Original images of all blots of LepR, GLUT4 and β-actin. The images of all blots for all replicates performed for LepR (100 kDa), GLUT4 (55 kDa) and β-actin (45 kDa). Figure S3 corresponds to the Western blot analysis shown in Figure 4A and 5A of main manuscript. n=6 per group. The red boxes represent the regions of the original blots used in main figures of manuscript.

Supplement figure 4

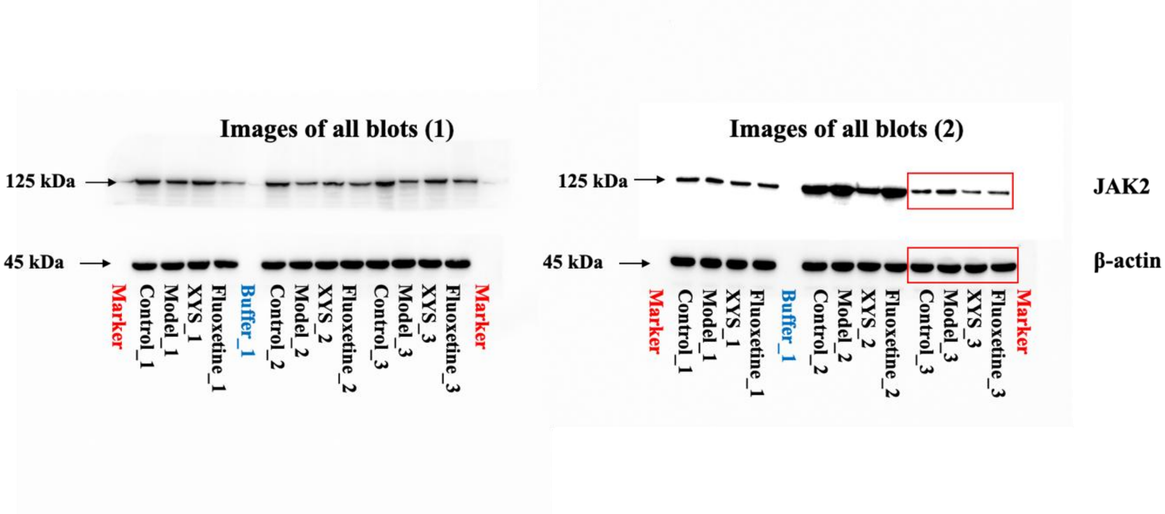

Figure S4. Original images of all blots of JAK2, and β-actin. The images of all blots for all replicates performed for JAK2 (125 kDa) and β-actin (45 kDa). Figure S4

corresponds to the Western blot analysis shown in Figure 5B of main manuscript. n=6 per group. The red boxes represent the regions of the original blots used in main figures of manuscript.

Supplement figure 5

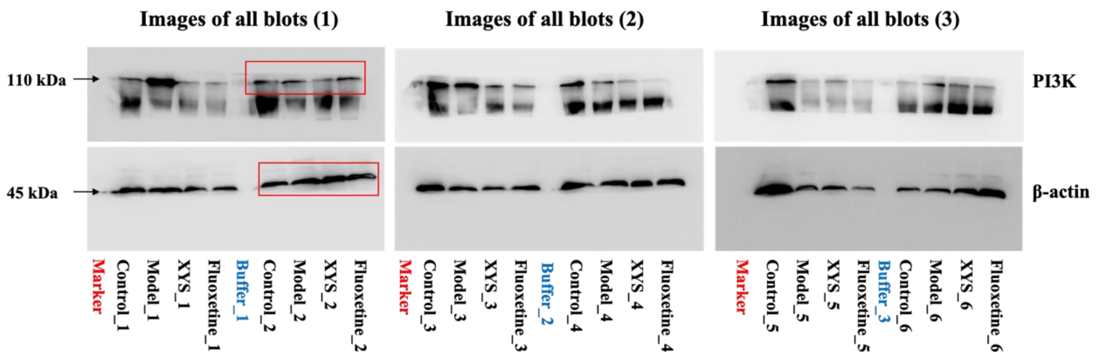

Figure S5. Original images of all blots of PI3K and β-actin. The images of all blots for all replicates performed PI3K (110 kDa) and β-actin (45 kDa). Figure S5 corresponds to the Western blot analysis shown in Figure 6B of main manuscript. n=6 per group. The red boxes represent the regions of the original blots used in main figures of manuscript.

Supplement figure 6

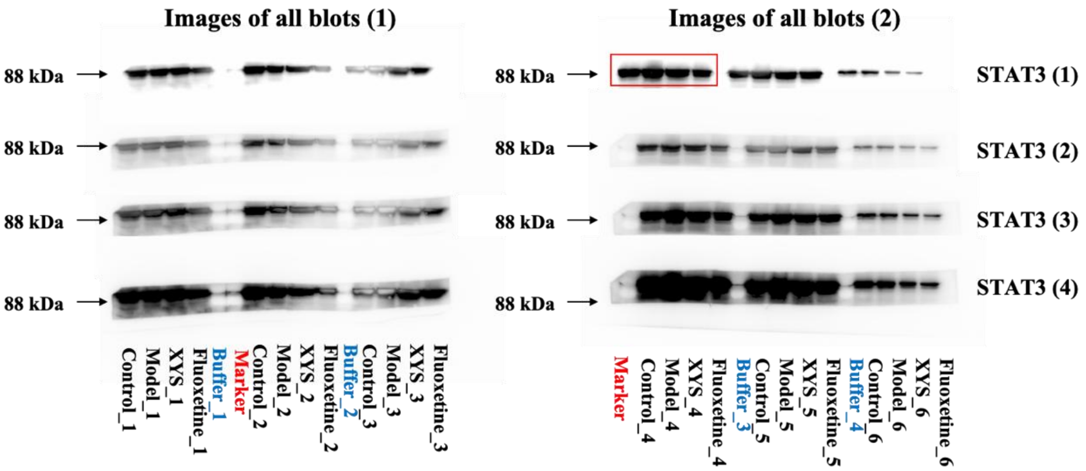

Figure S6. Multiple exposure images of STAT3. The STAT3 blots are overexposed in Figure S2, and multiple exposure images of STAT3 are shown as Figure S6. The red boxes represent the regions of the original blots used in main figures of manuscript.

Supplement figure 7

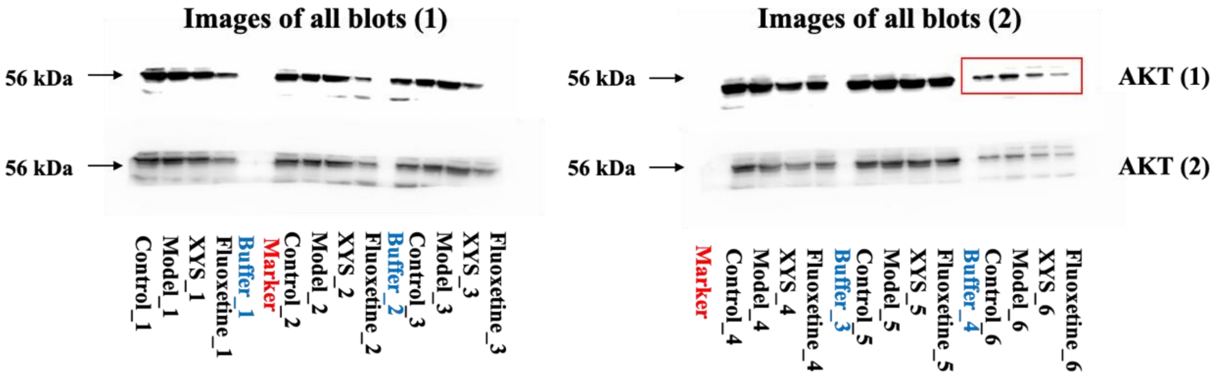

Figure S7. Multiple exposure images of AKT. The AKT blots are overexposed in Figure S2, and multiple exposure images of AKT are shown as Figure S7. The red boxes represent the regions of the original blots used in main figures of manuscript.

Supplement figure 8

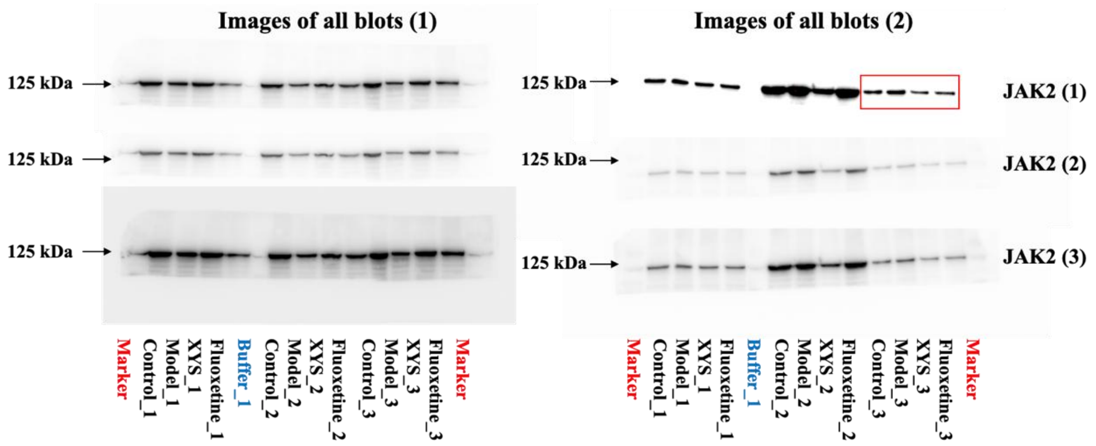

Figure S8. Multiple exposure images of JAK2. The JAK2 blots are overexposed in Figure S4, and multiple exposure images of JAK2 are shown as Figure S8. The red boxes represent the regions of the original blots used in main figures of manuscript.

Supplement figure 9

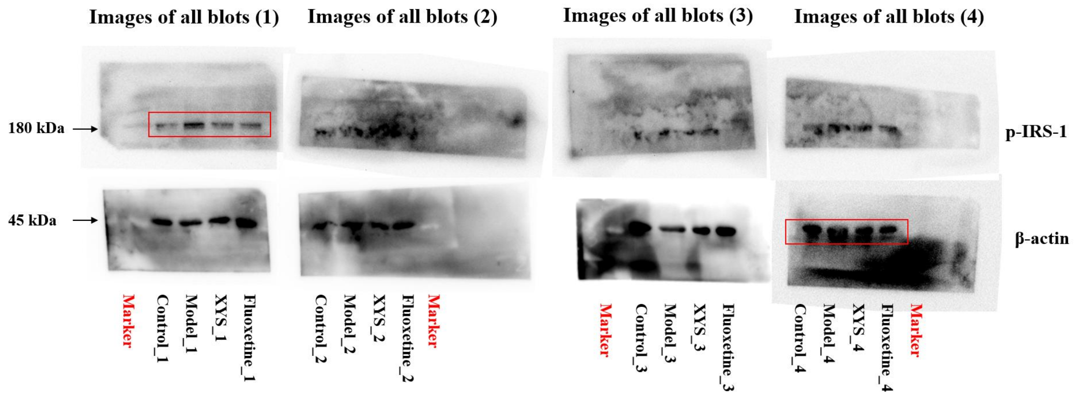

Figure S9. Original images of all blots of p-IRS-1, and β-actin. The images of all blots for all replicates performed for p-IRS-1 (180 kDa) and β-actin (45 kDa). Figure S9 corresponds to the Western blot analysis shown in Figure 6A of main manuscript. n=4 per group. The red boxes represent the regions of the original blots used in main figures of manuscript.

Supplement figure 10

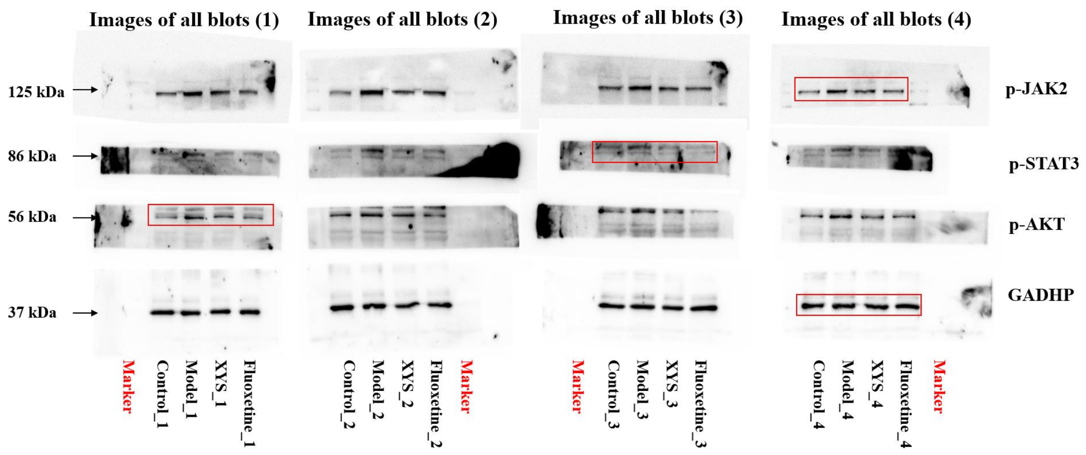

Figure S10. Original images of all blots of p-AKT, p-JAK2 and p-STAT3, and β-actin. The images of all blots for all replicates performed for p-JAK2 (125 kDa), p-STAT3 (86 kDa), p-AKT (56 kDa), and GADHP (37 kDa). Figure S10 corresponds to the Western blot analysis shown in Figure 5D, Figure 5C and Figure 6C of main manuscript. n=4 per group. The red boxes represent the regions of the original blots used in main figures of manuscript.
